# Teaching clinical reasoning in medical education: qualitative insights from educators at two UK universities

**DOI:** 10.1186/s12909-026-09037-6

**Published:** 2026-04-15

**Authors:** Claire Hemingway, George Lam, Laksha Bala, Ana V. Baptista, Pippa Watson, Matthew Jones, Mini Singh, Amir H. Sam

**Affiliations:** 1 Imperial College School of Medicine, Imperial College London, London, United Kingdom; 2 School of Medical Sciences, University of Manchester, Manchester, England, UK; 3 The Medical School, Edge Hill University, Ormskirk, United Kingdom

**Keywords:** Clinical reasoning, Curriculum, Faculty development, Medical educators, Undergraduate medical education

## Abstract

**Background:**

Deficiencies in clinical reasoning are a major contributor to diagnostic error and patient harm globally. There is increasing recognition of the need for longitudinal, integrated clinical reasoning teaching within medical curricula. This qualitative study explored educators’ understanding and perceptions of clinical reasoning at two UK medical schools with differing approaches to clinical reasoning education.

**Methods:**

Semi-structured interviews were conducted with 15 final-year medical clerkship supervisors across the two institutions. Interviews explored their understanding of clinical reasoning, experiences with teaching clinical reasoning, and perspectives on curriculum design and faculty development. Reflexive thematic analysis was used to identify key themes.

**Results:**

Educators described clinical reasoning as a complex cognitive operation. While they broadly shared an understanding of its core components, and its essential role in effective clinical decision making, their individual accounts revealed variations in scope and emphasis. Key themes included the need for longitudinally integrated curricula, explicit clinical reasoning instruction, structured faculty development, and challenges such as time constraints, institutional culture, and variable student engagement.

**Conclusion:**

The roles of a longitudinal and integrated curriculum design and faculty development in preparing educators to teach clinical reasoning were highlighted. These results are crucial for those considering new curriculum implementation, highlighting the need to design and introduce a focused faculty development programme for educators alongside the planned curriculum.

## Background

Clinical reasoning (CR) is the thinking and decision-making processes associated with clinical practice, involving both the cognitive operations of the clinician and interaction with the patient’s unique circumstances and the environment [1–3]. In practice, CR is the process by which clinicians observe, collect and interpret data to diagnose and treat patients [4]. In recent years, there has been increasing emphasis on CR given its contribution to diagnostic error and consequent patient harm worldwide [5].

Despite its critical role in diagnostic accuracy and patient safety, medical graduates feel unprepared in the domain of CR and enter clerkships with ‘poor to fair knowledge’ of CR concepts [6, 7]. This is due to the limited emphasis on teaching clinical reasoning principles and the lack of opportunity to practise comprehensive diagnostic thinking in current curricula [8].

There is a growing international consensus that undergraduate medical curricula should have a programme of explicit and longitudinal CR teaching [9, 10]. Some medical schools have responded by reforming their curriculum to implement longitudinal CR teaching throughout the undergraduate medical degree, but there is limited evidence on how to implement CR curricula in medical education [4, 11].

### Participating institutions and curricular approaches

To gain a broader understanding of educators’ perceptions of CR, this study was conducted at two UK medical schools (Institutions A and B) that feature different curricula. We describe the institutions’ curricula at the time of conducting this study.

At Institution A, the MBChB is a 5-year programme with voluntary opportunities to intercalate an additional year culminating in a Bachelor of Science (BSc). In 2016 the course was reformed to integrate CR education from year 2 into all parts of the curriculum. Current final-year students have experienced the full programme of integrated, longitudinal clinical reasoning education.

Institution B has a 6-year undergraduate medical programme with compulsory BSc intercalation. The comparable cohort of final-year medical students, in the curriculum that was in place at the time of the study, had received no formal CR teaching programme in preclinical years and had intermittent CR education during their final two years of studies. The curricular structure across these institutions were comparable, with two pre-clerkship years focused predominantly on basic sciences, followed by three rotational clerkship years in public-sector healthcare settings. The format of clinical reasoning teaching was, however, significantly different. Institution A had integrated CR principles and practice into both taught and experiential learning environment, aligned with formative and summative assessments in CR, whereas at Institution B, CR was introduced through intermittent explicit taught sessions during the last two clerkship years. Most CR learning was implicit from opportunistic experiential patient encounters.

Given the central role of educators in the effectiveness of CR instruction, and the requirement of a ‘critical mass’ of CR educators, it is essential to understand the factors influencing CR instruction [12–15]. The aim of this study was to explore the lived experiences and pedagogical challenges faced by medical educators when teaching CR within the undergraduate curriculum. By engaging with educators from diverse curricular backgrounds, we sought to address three primary research questions.

1. How do medical educators perceive and define clinical reasoning?
2. What curricular structures and universal barriers influence instruction in clinical reasoning?
3. What mechanisms of faculty support facilitate effective instruction in clinical reasoning?

We aimed to provide the wider medical education community with evidence-based insights into the challenges and facilitators of delivering undergraduate clinical reasoning teaching.

## Methods

### Ethical approval

The study received ethical approval from the Education Ethics Review Process at Imperial College London (Ref: EERP2223-019a). A qualitative approach using semi-structured interviews was conducted to facilitate an in-depth exploration of educators’ individual experiences and perceptions regarding teaching clinical reasoning.

### Participants

All participants were fully qualified specialists or primary care physicians, and held clerkship supervision responsibilities for final-year medical students at their respective medical schools. Final-year clerkship supervisors were specifically invited to participate because they work closely with students whose clinical reasoning skills are most advanced in the undergraduate curriculum. Potential participants were invited via email and in person, and selected through voluntary response sampling.

### Semi-structured interview design

Two researchers (CH, GL) conducted and recorded the 1:1 semi-structured interviews using Microsoft Teams. The interviews followed a series of prepared questions and prompts, developed and piloted by researchers before use in the main study. The interview guide was designed to provide a structured framework for participants to discuss key areas relevant to the study’s research questions. Both audio and video were recorded from the interviews and pseudonymised, but only audio recordings were subsequently transcribed. Participants were recruited until data saturation was reached. Repeat interviews were not conducted, and the transcripts were not returned to participants for comments or correction. This decision was made to reduce participant burden and because audio recordings captured by Microsoft Teams were sufficiently clear, minimising the likelihood of misinterpretation. The interview guide is included in Appendix 1. All interviews were conducted between 10th May 2023 and 7th July 2023.

### Data analysis

Data were collected anonymously and stored in accordance with the General Data Protection Regulation and Data Protection Act 2018. Transcripts were entered into NVivo 14.23.0 for qualitative thematic analysis. CH and GL performed the analysis, following an iterative and reflexive approach to identify and code common themes. Codes sharing similar meaning were amalgamated into subthemes and mapped into overarching major themes. Any discrepancies between the investigators were discussed and resolved through consensus to maintain the integrity of the analysis. To further enhance the validity and reliability of the thematic analysis, an independent third-party researcher (AVB) analysed the coded themes for triangulation.

Quotations were selected for inclusion in the Results section based on their typicality and representativeness of the identified sub-themes. To ensure a balanced perspective, the researchers verified that chosen excerpts accurately reflected the core sentiments expressed by multiple participants across both institutions, rather than representing outlier viewpoints.

### Research team and reflexivity

GL and CH are both fully registered clinicians in the United Kingdom and are clinical education fellows at Imperial College London. CH and GL were trained in qualitative research methods and received mentorship from experienced researchers. CH and GL were candid about their positions as clinical education fellows with all participants, and colleagues of the researchers were reassured that their participation would have no impact on professional relationships or evaluations. Efforts were made to create an atmosphere of trust and openness, where participants felt free to express their true thoughts and feelings. Any potential biases related to this relationship were discussed and addressed through reflexive practices and oversight from an independent third party (AVB), thereby enhancing the rigour of data collection and analysis.

## Results

**Fig. 1:**
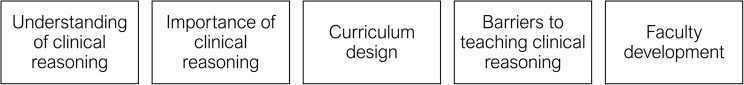
Five major themes inherent to the semi-structured interviews

Fifteen educators participated in semi-structured interviews (7 from Institution A and 8 from Institution B). Interview participants from Institution A and Institution B are shown in Table 1. The thematic analysis was guided by five major themes inherent to the semi-structured interview guide, which provided a framework for exploring educators’ experiences. The five major themes inherent to the semi-structured interviews are presented in Fig. 1.

**Table 1:** Interview participants by institution

Institution A	Institution B
E310	E298
E338	E302
E401	E352
E428	E749
E763	E683
E869	E818
E923	E849
	E880

Within these broad themes, specific coded concepts and subthemes emerged inductively from the data through the identification of common words and shared explicit or inferred meanings. Comparative insights identified the role of curriculum design and faculty development in preparing members of staff to teach CR.

### Major theme 1: understanding of clinical reasoning

Educators at both institutions shared a foundational understanding of clinical reasoning as the essential cognitive operations underpinning clinical diagnostic and management decisions. While individuals converged on the definition of CR as a structured ‘process’, their individual accounts revealed a layered understanding of cognitive processes (Table 2).

**Table 2:** Emergent subthemes and institutional alignment for Major Theme 1 (Understanding of clinical reasoning)

Major Theme	Emergent Subthemes	Institutional Alignment
1. Understanding of clinical reasoning	A. Information synthesis and decision making	Shared
	B. Cognitive models	Shared
	C. Metacognitive awareness	Shared

#### Subtheme 1A: information synthesis and decision making

While participants from both institutions offered distinct, personal definitions of CR, a shared conceptualisation emerged, describing CR as a process defined by the active collection and interpretation of clinical data to formulate a differential diagnosis and management plan.

*“When it’s teaching the students*,* we’d get them to think through the different diagnostic possibilities*,* probably try and work through and model how we think…”* (E401, A)

*“Clinical reasoning is using the available information we can glean from a patient’s story… to try to formulate a differential diagnosis“* (E302, B)

This definition illustrates that educators identify CR as the primary mechanism used to bridge the gap between initial clinical data collection and patient treatment in their own clinical practice.

#### Subtheme 1B: cognitive models

Participants at both institutions emphasised the importance of recognising cognitive operations involved in clinical practice. Participants described a cognitive model involving two distinct systems, one rapid, and one analytical.

*“Most broadly when I’m thinking about it*,* is this a pattern recognition approach or hypothetico-deductive reasoning approach?”* (E763, A)

*“You have to be very fast*,* and you have to be slow”* (E298, B)

This framing suggests that participants recognise the cognitive complexity involved in clinical practice. They acknowledge a personal need to navigate between ‘fast’ thinking and deliberate ‘slow’ analytical systems to ensure effective decision-making.

#### Subtheme 1C: metacognitive awareness

Educators across both institutions conceptualised clinical reasoning as an internal, metacognitive process which requires a continuous awareness of one’s own thinking patterns and potential cognitive biases, necessitating career-long refinement.

*“I think it’s to become more aware of clinical reasoning and things like cognitive bias as a form of medical error and diagnostic error.”* (E338, A)

*“We are teaching them how to think. And you can’t do that in a lecture or a course. That’s got to be a whole process. And it’s career-long*,* isn’t it? I’m still doing it.”* (E880, B)

These quotes demonstrate that educators perceive clinical reasoning as a continuous process. By reflecting on their own ongoing professional development and the persistent need for error-checking, they frame the monitoring of cognitive errors as an essential, lifelong requirement of clinical practice that remains relevant well beyond the completion of initial medical training.

### Major theme 2: importance of clinical reasoning

Thematic analysis demonstrated consensus regarding the importance of CR in medical education. Participants from both institutions articulated two primary reasons for the importance of CR education: mitigation of diagnostic error and managing uncertainty (Table 3).

**Table 3:** Emergent subthemes and institutional alignment for Major Theme 2 (Importance of clinical reasoning)

Major Theme	Emergent Subthemes	Institutional Alignment
2. Importance of clinical reasoning	A. Diagnostic safety and error mitigation	Shared
	B. Managing uncertainty	Shared

#### Subtheme 2A: diagnostic safety and error mitigation

Participants from both institutions identified clinical reasoning as a critical mechanism for minimising diagnostic error. Participants suggested that mistakes frequently arise not only from a deficit in medical knowledge, but also from a failure to engage in deliberate clinical reasoning, specifically the failure to regulate automatic thinking processes.

*“I see*,* mistakes happen because of the lack of the clinical reasoning.”* (E298, B)

*“We look at a patient*,* we think that’s what it is*,* and we persuade ourselves that that’s what it is without thinking it through. So*,* to me*,* clinical reasoning is about stopping*,* making sure that that first impression is a reasonable one*,* and then challenging it.”* (E869, A)

Participant E869(A)’s description of “stopping” and “challenging” illustrates a conscious effort to override intuitive responses with rigorous checks. In this context, the importance of CR lies in its function as a structured cognitive checkpoint, acting as an essential safeguard against the reasoning errors that lead to diagnostic error.

#### Subtheme 2B: managing uncertainty

A significant dimension of the importance of clinical reasoning is its role as a framework for navigating the inherent uncertainty in clinical practice. Participants from both institutions expressed that CR enables the clinician to move from theoretical assessment to action by quantifying uncertainty.

*“It will give them a bit more confidence to actually put in a management plan… instigating that initial management plan and doing it in a safe and sensible way.”* (E428, A)

*“I think*,* with new F1s [newly qualified doctors] to be really scared of actually doing the clinical reasoning… they’re terrified of committing to paper a diagnosis or a management plan.”* (E302, B)

These quotes suggest that the importance of clinical reasoning lies in its ability to facilitate decision making in the presence of uncertainty. While Participant E302(B) notes that novice clinicians are often “terrified” of the risk inherent in “committing to paper”, Participant E428(A) suggests that CR counters this fear by providing the “confidence” to act. In this context, CR provides the necessary intellectual scaffolding to ensure that management plans are not just actionable, but “safe and sensible”, allowing patient care to progress even when a diagnosis is not definitive.

### Major theme 3: curriculum design

Participants reflected that how CR is taught is just as crucial as the content itself. While analysis demonstrated a consensus regarding the importance of longitudinal integration, an institutional divergence emerged regarding the visibility and explicitness of CR delivery (Table 4).

**Table 4:** Emergent subthemes and institutional alignment for Major Theme 3 (Curriculum design)

Major Theme	Emergent Subthemes	Institutional Alignment
3. Curriculum design	A. Longitudinal and early integration	Shared
	B. Explicit vs. implicit delivery	Contrasting

#### Subtheme 3A: longitudinal and early integration

Despite differing curricular structures, participants converged in their belief that CR teaching should be longitudinal and integrated throughout the curriculum.

Participants at Institution B, with largely implicit, intermittent CR teaching in the curriculum, reflected that introducing these complex concepts as early as possible is beneficial to students’ CR development.

*“I think it could be better labelled and sign-posted*,* especially looking at things like some of the biases that students are probably introduced to a bit too late in the curriculum and they might benefit to doing earlier in the curriculum.”* (E352, B)

*“From a student point of view*,* I think it’s helpful to know that as early as possible. Rather than trying to learn something and then add it in later on.”* (E749, B)

Validating this approach, participants at Institution A expressed their longitudinally integrated curriculum allows their students to develop CR skills over time. E763(A) explicitly linked the curriculum design to essential professional skill development:

*“I know it’s integrated throughout and particularly through the clinical years… it’s something they are doing from an early age. So*,* yes*,* I do think [Institution A’s integrated approach] is probably a valuable approach that helps build some skills that are very important for them…”* (E763, A)

This comparison illustrates a shared pedagogical desire for CR to be an integrated theme that scaffolds students’ development of reasoning skills throughout their undergraduate education.

#### Subtheme 3B: explicit vs. implicit delivery

Participants highlighted contrasting perspectives regarding the visibility of clinical reasoning within their respective curricula. Participants at Institution A described CR as the explicit basis of their teaching framework, whereas educators at Institution B described it as an implicit element of clinical medicine.

CR teaching is explicitly integrated and emphasised at Institution A and this was recognised by study participants who apply this in their teaching practice. Educators at Institution A could identify the longitudinal, integrated nature of CR teaching and learning activities:

*“Now I think the whole thrust of them is about clinical reasoning problems*,* really*,* that they work through as a group with a facilitator.”* (E763, A)

*“…for each week*,* they have TCD sessions. Team case discussions… [they] go through various case scenarios and work out possibilities of differential diagnosis and management.”* (E310, A)

In addition, they emphasised the significance of CR in their curriculum, referencing alignment of activities to the integrated curriculum:

*“You need to know about clinical reasoning*,* because this is the whole basis of the curriculum right now*,* frankly*,* about how we want to teach our students.”* (E338, A)

In contrast, participants at Institution B perceived CR as a skill deeply embedded within clinical practice, often lacking formal recognition or ‘signposting’ in educational materials. Illustrating this implicit mindset, educators suggested that CR is indistinguishable from the general practice of medicine:

*“I think clinical reasoning is [part of the curriculum]… but that’s because it’s clinical medicine.”* (E849, B)

*“It always has been taught. If we’re talking about how you bring together history*,* examination and tests and formulate a differential and a management plan. If that’s your definition of clinical reasoning*,* then that’s how we’ve always done it as far as I’m aware.”* (E683, B)

Other participants reinforced this, noting that while clinical reasoning is inherent to their interactions with students, it is rarely explicitly identified as a distinct learning objective.

*“I think that it is a significant part*,* but it’s not labelled*,* signposted*,* as much as it should be.”* (E352, B)

*“I guess inherently*,* by discussing… Because if we’re assuming that clinical reasoning is the synthesis of information to arrive at a plan… I think that probably the students do get feedback*,* but it might not be labelled as feedback*,* then it’s not labelled as clinical reasoning.”* (E302, B)

Educators at Institution B reflected on the absence of this explicit structure. One participant highlighted the gap in formal instruction compared to experiential learning, expressing a desire for a more structured approach:

*“Just if there were formal teaching for students to teach them how to critically think*,* that would be absolutely amazing.”* (E298, B)

This comparison highlights that while CR occurs at both institutions, educators at Institution B feel they are relying on ‘implicit’ transmission of knowledge, whereas Institution A educators expressed support for an ‘explicit’ structured faculty development framework.

### Major theme 4: barriers to teaching clinical reasoning

Reflecting on their practices, participants from both institutions highlighted the barriers to consistent and sustainable CR teaching: time and service pressures, educational culture and faculty development opportunities, and student factors (Table 5).

**Table 5:** Emergent subthemes and institutional alignment for Major Theme 4 (Barriers to teaching clinical reasoning)

Major Theme	Emergent Subthemes	Institutional Alignment
4. Barriers to teaching clinical reasoning	A. Time and service pressures	Shared
	B. Educational culture and faculty engagement	Shared
	C. Student factors	Shared

#### Subtheme 4A: time and service pressures

Time constraints were the predominant barrier, impacting participants at multiple points in their teaching practice. Participants found it challenging finding time to extend their knowledge of CR and prepare for teaching sessions.

*“But even this once a week*,* it’s almost I had to fight to get that*,* and it is my spare time. They say*,* look*,* it’s your spare [time]. You want to do it for your own development; it’s up to you. Do you want to allocate it to students? It’s up to you.”* (E298, B)

Furthermore, participants from both institutions reported additional time required to convey the depth of CR concepts and thought processes. Some participants noted that time constraints can lead to a more superficial overview – for example, when students only observe reasoning practice.

*“You do need enough time as a tutor to unpack what you’re doing for either the students or the junior doctors to show your workings almost.”* (E401, A)

*“And [there are] time constraints as to explaining in a lot of detail*,* or whether it’s just a quick summary*,* or literally just them watching.”* (E749, B)

Heavy clinical workload compounds this issue, as participants noted that volume of patient care impacts educational opportunities for students.

*“We have 26 patients to see on a whole ward round so it can be tricky to do that as well as teach as well at the same time… That’s probably the biggest barrier.* (E428, A)

#### Subtheme 4B: educational culture and faculty engagement

Beyond practical constraints, participants expressed that the provision of training alone is insufficient without addressing deeper cultural barriers. At Institution A, despite the availability of resources, participants noted the challenge of achieving widespread methodological consistency. E923(A) predicted that clinical reasoning teaching would remain the domain of a specialised minority, while the wider workforce continued with traditional methods.

*“It’s always going to be [a] small*,* small group of people who are going to be trained who will be teaching clinical reasoning and others will be doing a traditional way of teaching.”* (E923, A)

Participants at Institution B shared these concerns but also highlighted the need for more integrated institutional support. Participants perceived a shortage of investment in their continued development, feeling that the necessary support structures to update their teaching practice could be improved.

*“It’s almost impossible for more senior consultants to really realistically do it. You have to decide whether an institution wants to invest [in] you.”* (E818, B)

#### Subtheme 4C: student factors

Student attitudes towards clinical reasoning were reported as a barrier to teaching CR. Participants discussed the challenge for students engaging in metacognitive processes and pausing to reflect and question their existing thoughts whilst reasoning.

***“****Getting people to stop and think is hard*,* and it’s hard concentrating on things and thinking about things and challenging things.”* (E869, A)

Furthermore, participants from both institutions noted that students were often exam focussed and prioritised exam preparation over understanding the deeper, theoretical aspects of CR.

*“Some of them are more focused towards their exams in the end. I don’t think that the students are keen to learn the clinical reasoning approach.”* (E923, A)

This assessment-driven engagement created a dichotomy in clerkship placements. One participant reported that engagement was high in activities focused on PACES (Practical Assessment of Clinical Evaluation Skills; a summative assessment) preparation, whereas they observed low engagement in formats that reflect clinical practice.

*“They also do a PACES ward round with one of my colleagues… and they love that… The difficulty is we don’t always see them engaging very much in those [routine] clinics.”* (*E818*,* B)*

#### Major theme 5: faculty development

Faculty development was recognised by participants as key in mitigating identified barriers and preparing educators to confidently teach CR. It can be defined as ‘any planned activity to improve an individual’s knowledge and skills in areas considered essential to the performance of a faculty member in a department or a residency programme’ [16]. Educators at both institutions were familiar with the concept of faculty development, although activities differed between institutions (Table 6).

**Table 6:** Emergent subthemes and institutional alignment for Major Theme 5 (Faculty Development)

Major Theme	Emergent Subthemes	Institutional Alignment
5. Faculty Development	A. Centralised and structured activities vs. self-directed	Contrasting
	B. Communities of practice	Contrasting
	C. Institutional visibility and communication	Contrasting

#### Subtheme 5A: centralised and structured activities vs. self-directed

Participants at Institution A reported attendance at regular workshops focused on clinical reasoning, and annual educator ‘away days’ including updates on CR at their institution:

*“And they have invested heavily in teaching us to use it. There’s trainer development sessions that run several times a year which focused on teaching clinical reasoning using the model.”* (E401, A)

Participants at Institution B also attend faculty development activities, yet in contrast to Institution A, participants reported these activities were not designed with a specific focus on CR.

*“I’ve never done anything specific on clinical reasoning*,* that’s why I said I’d quite like to learn more about how it’s taught.”* (E818, B)

This perceived gap has led to a more self-directed rather than institution-led approach, with another participant advocating for proactive learning beyond institutional materials:

*“Don’t rely only on the material which you get. Just do it in your time. Do something different.”* (E298, B)

Institution A’s wider focus on clinical reasoning and targeted CR faculty development programme was influential on their educators. Participants from Institution A reported an understanding of the curricula objectives from faculty development activities:

*“In that*,* there is a lot of information to teach*,* from the way the curriculum [is structured] to what is the learning methodologies and things*,* are there on the website so we can go and read in that.”* (E923, A)

Furthermore, educators at Institution A expressed convergent understanding of how to implement teaching and learning activities to achieve these outcomes. ‘Away Days’ were stated to be the most valuable faculty development for participants who experienced them. Most educators at Institution A mentioned these, using shared language to explain the structure of these days and take-home points.

*“So the away day is… it’s one full day… There will be multiple things included in that day. And then the afternoon will be much more about workshops*,* depending on what your role is. So one of those will be*,* for example*,* clinical reasoning.”* (E338, A)

*“The more I know about it*,* then I can teach them in a better way. I can organise the way. Some of the information is self-taught*,* self-learnt in the clinical reasoning. Some of them I learnt through the away day”* (E923, A)

#### Subtheme 5B: communities of practice

Participants at both institutions highlighted the need for planned activities, where they can meet with colleagues and faculty to build a community of practice that crosses traditional healthcare service boundaries (community and hospital). Educators from Institution A felt their faculty development structure enabled sharing of best educational practice:

*“It’s a good way of getting some chance to meet secondary care clinicians. I’ve been to some really excellent things at that.”* (E763, A)

At Institution B, where such forums were not yet established, educators expressed a desire for more structured dialogue. Participants noted that specific conversations regarding the pedagogy of clinical reasoning were less frequent. Educators suggested that formalising these networks would be a valuable addition to their professional development.

*“I think part of it is actually not having that much conversation about it [clinical reasoning]. I think even amongst clinicians… Actually*,* it would be maybe helpful to have a bit more about that*,* from a professional development point of view*,* certainly.”* (E749, B)

#### Subtheme 5C: institutional visibility and communication

Finally, participants identified that the visibility of faculty development played a crucial role in engagement. At Institution A, participants reported that clinical reasoning was kept salient through frequent, targeted communication. This constant signalling served not only to advertise events but to reinforce the institutional priority of CR instruction.

*“Most of the emails and other communications have been focusing on clinical reasoning.”* (E923, A)

In contrast, educators at Institution B noted that a lack of communication contributed to the invisibility of training opportunities. Despite a willingness to learn, participants reported that information regarding development sessions often failed to reach them, creating a barrier to access that was logistical rather than motivational.

“But I never see… I don’t get much information about these things. I don’t know why. I would love to.” (E298, B)

Overall, faculty development was reported to give confidence and clarity to educators, positively enhancing their approach to teaching CR.

## Discussion

Several overarching themes emerged that provide a nuanced view of how educators understand and teach clinical reasoning at their respective institutions.

### Understanding and importance of CR

Participants from both institutions described clinical reasoning using a range of interrelated concepts, including cognitive operations, dual process theory and reflective self-awareness of thinking. While these accounts reflect a broadly shared orientation toward CR as the cognitive work underpinning diagnostic and management decisions, the variation in emphasis and scope suggests that understandings are shaped by differing contextual and professional perspectives. These findings resonate with the conclusions of Young et al., who argue that surface-level consensus of clinical reasoning definitions may mask deeper conceptual boundaries within individuals’ educational and clinical practice [17].

Participants indicated the key relationship between cognitive factors and diagnostic error. This is substantiated by empirical studies and a monograph on Diagnostic Errors from the World Health Organisation [18, 19]. It has been argued that diagnostic error can arise from both analytical and pattern recognition thinking and that multiple interventions play a role in mitigating cognitive errors in diagnosis [18–20]. Our findings support this, with participants expressing the need for formalised CR teaching, enabling students to develop cognitive frameworks and reflective practice to mitigate diagnostic errors.

### Curriculum design

Our findings demonstrate an institutional variance in the teaching and integration of clinical reasoning within the medical curriculum, despite the universal agreement on its foundational importance. At Institution A, CR is deeply embedded into the curriculum in an explicit way, aligning with the faculty’s training. This holistic approach mirrors the emphasis on a comprehensive, integrated curriculum advocated by consensus statements of educators in the United Kingdom and the United States [1, 7].

In contrast, the participants at Institution B, while valuing the skill, observed the absence of a structured pedagogical framework for CR. Specifically, they mention that topics such as cognitive biases are “introduced a bit too late in the curriculum” *(E352*,* B)* and call for instruction to begin earlier in the course. This concern is not unique to Institution B; an international survey of 313 medical education experts revealed that only 28% of respondents report the presence of a longitudinal CR curriculum, despite 85% stating such a curriculum was needed [10].

Our analysis highlights that educators view implicit learning as insufficient. Participant E298(B) called for more “formal teaching”, which indicates a recognition that experiential exposure alone does not equip students with the necessary CR frameworks. This supports the argument that curricula should move to explicit CR instruction. This aligns with recent recommendations from a European consortium of health professions educators, which advocate for clinical reasoning to be made explicit in national learning objective catalogues to challenge the misconception that reasoning cannot be taught [21]. Furthermore, in the UK context, the General Medical Council’s ‘Outcomes for Graduates’ mandates that new doctors must be able to explain clinical reasoning in action and justify their selection of investigations [22]. Reliance on implicit CR instruction may be insufficient to ensure that all graduates meet the regulatory standard for explicit competency.

### Barriers to teaching CR

Even though educators recognise the importance and value of teaching clinical reasoning, there are significant barriers to implementing focused CR curricula. These include factors such as time, culture and faculty expertise [7, 10, 15]. Our cross-institutional data provides novel insights, highlighting perceived barriers at Institution B and lived experience of barriers experienced by educators with an integrated curriculum. Participants in both institutions identified time as the greatest barrier to teaching CR, supporting previous findings [7, 10, 15]. Our study responds to the call by Sudacka et al. for exploration of perceptual differences to these barriers, delineating how time pressures impact educators [15]. Participants felt their time was limited at multiple stages of teaching: when preparing for sessions, when engaging in continuing professional development (CPD) to extend their knowledge of CR and when teaching in clinical practice. Educators in our study recognised the additional time pressure introduced by their ‘double competencies’, being expected to fulfil the role of a skilled clinician and effective teacher simultaneously [15]. The pressure of balancing these responsibilities was also reported as a limitation to workforce, supporting the model proposed by Sudacka et al. of a ‘vicious cycle’ in the development of a ‘critical mass’ of CR educators [15]. The consistency with which time is seen as a constraint to the clinical educator highlights the low value teaching has in healthcare, and yet without it, one cannot have a safe and competent workforce. This tension may be addressed by rethinking approaches to medical education, integrating it into healthcare policy and quality management systems on par with health outcomes. With respect to the latter, systematic accountability for funding of medical education for those who are tasked with providing clerkship experiences is critical.

Lack of faculty expertise and training in clinical reasoning has previously been identified as a significant barrier to implementation [10]. Building on this, our study provides novel insights by showing that some educators perceive CR education as non-traditional and believe additional training is required to teach it effectively. Further work is therefore needed to challenge the perception of CR as a niche activity rather than a foundational component of medical education.

### Faculty development

Our results reinforce the role of faculty development to train educators and enable curriculum change [23]. Participants at both institutions echoed existing literature by emphasising the importance of faculty development, while cross-institutional insights highlighted it as fundamental to the successful implementation of a longitudinal and integrated CR curriculum [10, 15].

We identified the ‘Away Day’ at Institution A as an effective faculty development programme. The structured and well-resourced nature of this programme, noted by participants, reflects a system-theory-based approach to delivering large-scale faculty development, as detailed by Singh et al. [23]. The annual ‘Away Day’ at institution A is offered free of charge to all clinician educators and complements other synchronous and asynchronous faculty development opportunities provided as part of a comprehensive suite of CPD material. The structure of a typical ‘Away Day’ allows medical school faculty to deliver priority messages (for example, relating to CR teaching) to the entire educator cohort in plenary sessions, whilst also giving clinicians a broad choice of workshop activities to align with individual development needs. This faculty development programme aligns well with Kirkpatrick’s model of training programme evaluation [24]. Participants from Institution A were engaged and interested in faculty development, and “looking forward” to future Away Days. Educators were able to highlight key learning points and outlined how faculty development has influenced their CR educational practice.

Furthermore, our study highlights the crucial involvement of medical school faculty in faculty development. Most of the training received for educational roles relates to postgraduate teaching [25]. Educators may therefore find CPD opportunities in their clinical field but not related to medical education or clinical reasoning. Staff involved in curriculum design at Institution A also designed dedicated CR faculty development for educators. These ‘Away Days’ are offered to all clinicians involved in teaching students from Institution A, in primary and secondary care. Our analysis highlighted that educators valued these faculty development opportunities for enabling interaction with teachers from diverse healthcare settings, which facilitated the sharing of ideas and exploration of key CR concepts. Through this coordinated faculty development, educators cross monodisciplinary boundaries.

By contrast, educators at Institution B reflected on the lack of ‘conversation’ around clinical reasoning. Although educators were interested in CR-related CPD, they found access to institutional faculty development a challenge. Educators from Institution B were instead highly motivated in pursuing individual CR activities to extend their knowledge – for example, through attending online courses. Although valuable, educators may miss out on communities of practice developed through group workshops. As de Carvalho-Filho et al. describe, communities of practice established through faculty development can strengthen institutional learning cultures and support the empowerment and development of educators [26].

Institution A’s programme provides empirical evidence for this, with educators reporting that faculty “have invested heavily in teaching us” and reflecting positively on meeting together during faculty development.

Finally, our findings highlight the importance of communicating curricular priorities. Consistent and targeted communication at Institution A reinforced CR as an organisational priority, suggesting that even a motivated workforce requires clear institutional signposting to engage with developmental opportunities.

### Limitations

Participants were recruited to the study through convenience sampling, with invitations sent through approved medical school communication channels and at faculty development meetings. This method could introduce a selection bias towards potentially attracting educators who are more actively engaged or have stronger opinions regarding teaching clinical reasoning. All participants were clinician educators working across two regions in the UK; perspectives from other geographical areas may yield new insights into the experiences and perceptions of teaching clinical reasoning to medical students. The findings are based on educators’ self-reported experiences, perceptions and practices. Such data can be subject to recall bias or social desirability bias, despite efforts to create an open and trusting interview environment [27].

A further limitation is the lack of granular demographic data regarding the participants’ specific years of clinical and teaching experience. While our inclusion criteria ensured a baseline level of seniority, we did not record individualised professional histories. Consequently, we are unable to determine the extent to which varying levels of personal experience, as opposed to institutional curriculum structure, influenced the educators’ perceptions of clinical reasoning.

Furthermore, we acknowledge that this study captures the perceptions and experiences of educators rather than objective measures of student competence. While educator insights are foundational to curriculum delivery, they do not provide empirical evidence of learning. Future research should aim to evaluate these curricula at higher levels of educational impact, such as focusing on behavioural changes in clinical practice and the long-term influence on patient safety outcomes.

## Conclusion

In this paper, we have presented cross-institutional insights into clinical reasoning curricula at two UK medical schools. The data presented here reinforces the notion that medical educators, regardless of institutional background, prefer longitudinal and integrated curricula to teach CR. We provide greater exploration of barriers faced by educators when teaching CR and give empirical evidence for the role of faculty development in effective implementation of integrated CR curricula. Targeted faculty development may enable a valued community of practice, shared faculty priorities and clarity when teaching CR. These results are crucial for those considering new curriculum implementation, highlighting the need to design and introduce a focused faculty development programme for educators alongside the planned curriculum. Future research could be undertaken to explore the perceptions and understanding of students at institutions with different CR curricular structures. Extensions of this work beyond final-year medical students would be valuable to assess the impact on early-year clinicians’ reasoning in practice.

## Data Availability

The datasets used and/or analysed during the current study are available from the corresponding author on reasonable request.

## Appendix 1

### Semi-structured interview guide

*Prompt for facilitator: Introduce educator to semi-structured interview*,* purpose to explore clinical reasoning concepts*,* perceptions and faculty development*

### Understanding/knowledge of CR

1. What do you understand by the term clinical reasoning (CR)?
  - *Could you provide a definition of CR? Name any potential tools to guide clinical reasoning.*

2. Explain to me how you make decisions in your clinical practice.
  - *Do you apply clinical reasoning concepts? How do you interpret physical examination findings? How do you choose and interpret diagnostic tests? How do you formulate differential diagnoses?*

3. What factors influence your clinical reasoning?

### Medical school teaching of CR

1. Let’s discuss clinical reasoning teaching in the curriculum at your medical school.
  - *What sessions are there? How did they work? What was your involvement in the sessions? Do you think this teaching is effective/valuable for students?*

2. Is clinical reasoning a significant part of your curriculum? Which part of the clinical reasoning curriculum has been most helpful to your students’ learning, and in what ways/why?
  - *What are the most useful parts of the course?*

### Application of CR

1. How often do you see students applying clinical reasoning in practice?
  - *Give us examples of when you’ve seen it used in practice – did students apply their classroom learning? How often do you give feedback to students on their clinical reasoning abilities in practice?*

2. Do you try and improve your own clinical reasoning skills?
  - *If so*,* how? Any evidence of CPD activities? Additional courses?*

3. Do you undertake any faculty development activities, within your medical school for in clinical reasoning?
  - *If so, please outline these activities. Would educators like any more faculty development than they are currently receiving?*

### Perceptions/confidence of CR

1. Do you feel it is important to be taught how to clinically reason?
2. How confident are you teaching concepts of clinical reasoning?
  - *How confident are your students?*

3. How do you feel clinical reasoning teaching will influence your students as foundation year doctors?

### Concluding

1. Barriers to clinical reasoning teaching.
  - *What are the barriers to learning clinical reasoning for your own professional development? What factors prevent this?*
  - *What are the barriers to implementing clinical reasoning teaching?*
2. Is there anything you would like to say that has not been covered

*Prompt for facilitator: Thank educators for participation*

## Abbreviations

CR: Clinical reasoning

## References

[1] Cooper N, Bartlett M, Gay S, Hammond A, Lillicrap M, Matthan J, et al. Consensus statement on the content of clinical reasoning curricula in undergraduate medical education. Med Teach. 2021;43(2):152–9. 33205693 10.1080/0142159X.2020.1842343

[2] Daniel M, Rencic J, Durning SJ, Holmboe E, Santen SA, Lang V, et al. Clinical reasoning assessment methods: a scoping review and practical guidance. Acad Med. 2019;94(6):902–12. 30720527 10.1097/ACM.0000000000002618

[3] Elvén M, Welin E, Edström DW, Petreski T, Szopa M, Durning SJ, et al. Clinical reasoning curricula in health professions education: a scoping review. J Med Educ Curric Dev. 2023;10:1–15. 10.1177/23821205231209093 PMC10605682 37900617

[4] Singh M, Collins L, Farrington R, Jones M, Thampy H, Watson P, et al. From principles to practice: embedding clinical reasoning as a longitudinal curriculum theme in a medical school programme. Diagnosis. 2022;9(2):184–94. 10.1515/dx-2021-0031 34256424

[5] Graber ML, Kissam S, Payne VL, Meyer AND, Sorensen A, Lenfestey N, et al. Cognitive interventions to reduce diagnostic error: a narrative review. BMJ Qual Saf. 2012;21(7):535–57. 10.1136/bmjqs-2011-000149 22543420

[6] Monrouxe LV, Grundy L, Mann M, John Z, Panagoulas E, Bullock A, et al. How prepared are UK medical graduates for practice? A rapid review of the literature 2009–2014. BMJ Open. 2017;7:e013656. 10.1136/bmjopen-2016-013656 PMC5253586 28087554

[7] Rencic J, Trowbridge RL, Fagan M, Szauter K, Durning SJ. Clinical reasoning education at US medical schools: results from a national survey of internal medicine clerkship directors. J Gen Intern Med. 2017;32(11):1242–6. 28840454 10.1007/s11606-017-4159-y PMC5653563

[8] Han H, Roberts NK, Korte R. Learning in the real place: medical students’ learning and socialization in clerkships at one medical school. Acad Med. 2015;90(2):231–9. 25354072 10.1097/ACM.0000000000000544

[9] Graber ML, Rencic J, Rusz D, Papa F, Croskerry P, Zierler B, et al. Improving diagnosis by improving education: a policy brief on education in healthcare professions. Diagnosis. 2018;5(3):107–18. 30145580 10.1515/dx-2018-0033

[10] Kononowicz AA, Hege I, Edelbring S, Sobocan M, Huwendiek S, Durning SJ. The need for longitudinal clinical reasoning teaching and assessment: results of an international survey. Med Teach. 2020;42(4):457–62. 32017640 10.1080/0142159X.2019.1708293

[11] Hawks MK, MacIuba JM, Merkebu J, Durning SJ, Mallory R, Arnold MJ, et al. Clinical reasoning curricula in preclinical undergraduate medical education: a scoping review. Acad Med. 2023;98(8):958–65. 36862627 10.1097/ACM.0000000000005197

[12] Boileau E, Audétat MC, St-Onge C. Just-in-time faculty development: a mobile application helps clinical teachers verify and describe clinical reasoning difficulties. BMC Med Educ. 2019;19(1):120. 31039779 10.1186/s12909-019-1558-2 PMC6492340

[13] Addy TM, Hafler J, Galerneau F. Faculty development for fostering clinical reasoning skills in early medical students using a modified Bayesian approach. Teach Learn Med. 2016;28(4):415–23. 27283028 10.1080/10401334.2016.1186551

[14] Weinstein A, Gupta S, Pinto-Powell R, Jackson J, Appel J, Roussel D, et al. Diagnosing and remediating clinical reasoning difficulties: a faculty development workshop. MedEdPORTAL. 2017;13:10650. 30800851 10.15766/mep_2374-8265.10650 PMC6338136

[15] Sudacka M, Adler M, Durning SJ, Edelbring S, Frankowska A, Hartmann D, et al. Why is it so difficult to implement a longitudinal clinical reasoning curriculum? A multicenter interview study on the barriers perceived by European health professions educators. BMC Med Educ. 2021;21:575. 34772405 10.1186/s12909-021-02960-w PMC8588939

[16] McLean M, Cilliers F, van Wyk J. Faculty development: yesterday, today and tomorrow. Med Teach. 2008;30(6):555–84. 18677659 10.1080/01421590802109834

[17] Young M, Thomas A, Lubarsky S, Ballard T, Gordon D, Gruppen LD, et al. Drawing boundaries: the difficulty in defining clinical reasoning. Acad Med. 2018;93(7):990–5. 29369086 10.1097/ACM.0000000000002142

[18] Zwaan L, Thijs A, Wagner C, van der Wal G, Timmermans DRM. Relating faults in diagnostic reasoning with diagnostic errors and patient harm. Acad Med. 2012;87(2):149–56. 22189886 10.1097/ACM.0b013e31823f71e6

[19] Sheikh SA, Donaldson L, Dhingra-Kumar N, Bates D, Kelley E, Larizgoitia I, et al. Diagnostic errors: technical series on safer primary care. Geneva: World Health Organization; 2016.

[20] Norman GR, Eva KW. Diagnostic error and clinical reasoning. Med Educ. 2010;44(1):94–100. 20078760 10.1111/j.1365-2923.2009.03507.x

[21] Hege I, Adler M, Donath D, Durning SJ, Edelbring S, Elvén M, et al. Developing a European longitudinal and interprofessional curriculum for clinical reasoning. Diagnosis. 2023;10(3):218–24. 36800998 10.1515/dx-2022-0103

[22] General Medical Council. Outcomes for graduates. London: General Medical Council. 2020. Available from: https://www.gmc-uk.org/education/standards-guidance-and-curricula/standards-and-outcomes/outcomes-for-graduates.

[23] Singh M, Grundy J, Mirza MO, Ramani S. Twelve tips to deliver large-scale faculty development in health professional education: a system-based approach. Med Teach. 2024;47(4):603–9. 39330212 10.1080/0142159X.2024.2407123

[24] Kirkpatrick DL. Evaluating training programs: the four levels. San Francisco (CA): Berrett-Koehler; 1994.

[25] Norman RI, Dogra N. A survey of the practice and experience of clinical educators in UK secondary care. BMC Med Educ. 2014;14:229. 25338782 10.1186/1472-6920-14-229 PMC4287536

[26] de Carvalho-Filho MA, Tio RA, Steinert Y. Twelve tips for implementing a community of practice for faculty development. Med Teach. 2020;42(2):143–9. 30707855 10.1080/0142159X.2018.1552782

[27] Althubaiti A. Information bias in health research: definition, pitfalls, and adjustment methods. J Multidiscip Healthc. 2016;9:211–7. 27217764 10.2147/JMDH.S104807 PMC4862344

